# Physical activity during pregnancy and maternal-child health (PAMELA): study protocol for a randomized controlled trial

**DOI:** 10.1186/s13063-015-0749-3

**Published:** 2015-05-24

**Authors:** Marlos Rodrigues Domingues, Diego Garcia Bassani, Shana Ginar da Silva, Carolina de Vargas Nunes Coll, Bruna Gonçalves Cordeiro da Silva, Pedro Curi Hallal

**Affiliations:** Postgraduate Programme in Physical Education, Federal University of Pelotas, Rua Luís de Camões, 625, 96055630 Pelotas, Brazil; GEEAF - Physical Activity Epidemiology Research Group, Rua Luís de Camões, 625, 96055630 Pelotas, Brazil; Centre for Global Child Health, The Hospital for Sick Children, 555 University Avenue, Toronto, ON M5G 1X8 Canada; Department of Paediatrics, Faculty of Medicine, University of Toronto, 1 King’s College Circle, Toronto, ON M5S 1A8 Canada; Postgraduate Programme in Epidemiology, Federal University of Pelotas, Rua Marechal Deodoro, 1160, 96020220 Pelotas, Brazil

**Keywords:** Exercise, Motor activity, Randomized controlled trial, Pregnancy, Infant premature, Cohort studies, Newborn, Gestational diabetes, Pre-eclampsia, Postpartum depression

## Abstract

**Background:**

Preterm birth is associated with most cases of neonatal deaths and negative health outcomes, and hypertensive disorders. Hypertension is influenced by maternal behavior, such as physical activity. Physical activity is associated with better outcomes for mother and fetus, besides healthier weight gains during pregnancy. Few women are physically active during pregnancy and few clinical trials have been carried out with pregnant women. The aim of this paper is to describe the protocol of a controlled trial evaluating whether regular exercise during pregnancy may result in improved maternal-child health and neonatal outcomes.

**Methods/Design:**

The PAMELA (Physical Activity for Mothers Enrolled in Longitudinal Analysis) trial is a randomized controlled trial nested in a birth cohort study. Eligible women belonging to the birth cohort will be invited (between the 16th and 20th week of gestation) to enroll in the trial. Baseline data (blood and urine samples, anthropometry and pulmonary function) will be collected at enrollment. The same assessments will be repeated eight and 16 weeks after baseline. After randomization, women will be allocated into either one of these groups: control, 426 women who will be advised to keep their usual daily activities; and intervention, 213 women who will engage in an exercise program, three sessions a week. At least 70 % attendance over 16 weeks will be required to be considered compliant to the intervention. Exercise protocol will include aerobics, strength and flexibility training. Maternal and child outcomes will be measured at the 36th week of gestation, at birth and at three, 12, 24 and 48 months postpartum. An intention-to-treat analysis will be performed.

**Discussion:**

Few women are active during pregnancy and a vast majority decrease their activities or even quit exercising. We present a population-based regular exercise intervention focused on the prevention of hypertension, pre-eclampsia and preterm birth. Data on the underlying cohort will allow future analysis using different outcomes with low probability of recall bias or misclassification of exposure status. Results will potentially influence prenatal care counseling in regards to physical activity.

**Trial registration:**

Clinicaltrials.gov identifier: NCT02148965, registered on 22 May 2014.

## Background

The World Health Organization estimates that over 10 % of births worldwide are preterm [1]. Based on previous population studies in the city of Pelotas (Brazil), the preterm birth rate is increasing (from 6.3 to 14.7 % between 1982 and 2004) [2]. Preterm birth is associated with most cases of neonatal deaths and negative consequences throughout childhood and adult life [3, 4].

Among the strongest predictors of preterm birth are hypertensive disorders of pregnancy [5–7]. Evidence suggests that preterm birth and gestational hypertension risk may be altered by regular leisure-time physical activity [8–12]. The potential hypothesis for such a mechanism is based on reduction in blood pressure, improvement in blood lipids profile, reduced oxidative stress and inflammation reduction [13–15]. Studies also have shown that leisure-time physical activity during pregnancy is associated with lower risk of excessive weight gain [16] and better psychological health [17, 18]. Although evidence supports the benefits of physical activity during pregnancy, few women in Brazil are active during gestation, and the level of leisure-time activity decreases as pregnancy advances [19]. Further, the effect of physical activity on mother-child health outcomes is not fully understood.

With respect to different outcomes, such as diabetes, trials during pregnancy do not provide enough evidence that exercise is effective [20]. The main goal of conducting a trial in the Brazilian population is because physical activity among Brazilian women is highly associated with socioeconomic status and other characteristics not easily controlled statistically during analysis. This trial will allow for the balance of such potential confounders, as we are using a population-based sample and random allocation. Moreover, few experimental studies have been carried out to study such associations in large samples.

### Rationale

Although early studies on the effects of leisure-time physical activity during pregnancy were concerned about potential harmful effects to the health of the mother and the fetus, these have not been proven over time [21]. Current guidelines suggest that pregnant women should engage in at least 30 min of moderate-intensity physical activity on most, if not all days of the week, in the absence of medical or obstetric complications, in agreement with the recommendations for healthy adults [22].

Today, there is growing evidence supporting the association of safe physical activity during pregnancy with benefits to maternal and child health [23, 24]. However, most of the scientific evidence derives from observational studies, and there is a need for well-designed experimental studies that enable a better understanding on the impact of exercise during pregnancy on various maternal and mother-child health outcomes.

### Aim

The aim of this study is to present the experimental protocol of a trial aimed at evaluating the effectiveness of an exercise intervention, by comparing the intervention and control groups with infant outcomes assessed later in life. Data will be collected on: prematurity, gestational age, gestational weight gain, blood pressure, fasting blood glucose, postpartum weight retention, postpartum depression and birth weight.

## Methods/Design

### Study design

A randomized controlled trial will be carried out and eligible women will be sampled from the 2015 Pelotas (Brazil) Birth Cohort Study. Currently three birth cohorts (1982, 1993 and 2004) are ongoing in the city of Pelotas (southern Brazil), each with more than 4,000 subjects that were enrolled soon after birth (at hospital). The 2015 Pelotas Birth Cohort Study recruits pregnant women from health services to begin gathering data prospectively during the prenatal period. More than 3,500 women are expected to be included in the study, providing information on several health-related aspects. All women with an expected delivery date from 1 January to 31 December 2015 are eligible to be included in the cohort.

### Ethical considerations

The clinical trial protocol and the 2015 Pelotas Birth Cohort Study were submitted to the Physical Education School Ethics Committee and were approved under the numbers 649.244 and 522.064, respectively. Also, the following procedures will be followed: Participation in the study will only occur only after reading and signing the consent form; All women will be guaranteed the right to not participate in the study; We will ensure confidentiality of the collected data and document numbers (identifications, used to link databases); and Women presenting health problems during the study will be referred to appropriate health services.

The study is also registered on the Clinicaltrials.gov website under the registry number NCT02148965, entitled’Effects of exercise during pregnancy on maternal and child health: a randomized clinical trial (PAMELA)’.

### Recruitment and participants

**Fig. 1:**
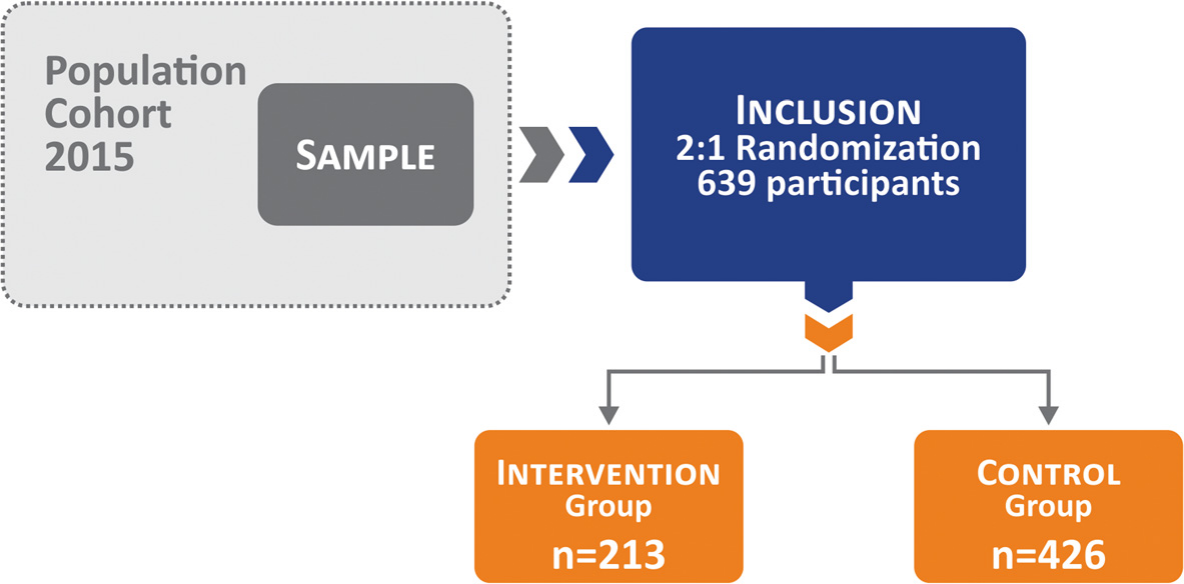
Study design

All locations where pregnant women would potentially seek assistance during pregnancy, such as medical laboratories, ultrasound clinics, polyclinics, public primary health units, hospitals, university clinics and private obstetric and/or gynecological clinics, are being visited daily since April 2014 to identify eligible women. Based on the first prenatal interview, those women who meet inclusion criteria will be invited (by a standard phone contact) to participate in the randomized controlled trial. The recruitment will continue during the prenatal study until we achieve the required sample size. Fig. 1 displays the study design.

### Randomization

Pregnant women meeting the inclusion criteria and who agree to participate in the randomized controlled trial will be allocated randomly into either the intervention or control group. The randomization process will be done in blocks of nine women using random software sampling. Each block will therefore result in the allocation of three women for the intervention and six women for the control group, ensuring a recruitment balance of 1:2 throughout the study. This ratio was chosen as it would minimize costs and behavioral changes in women from the remaining cohort, whilst assuring that statistical power would be unaffected. As the cohort is an observational study, our option was to expose the lowest possible number of women to the intervention. After randomization, women will be allocated into either one of these groups: control, 426 women who will keep their usual daily activities; and intervention, 213 women who will engage in a physical activity program, three sessions a week.

### Exclusion criteria

During the first contact, women will be interviewed about several aspects of their overall health, and those presenting any of the conditions described in the Table 1 will not be eligible for the trial.

**Table 1:** Exclusion criteria of the randomized controlled trial

Exclusion criteria	Description
Age	Below 18 years
Hypertension	Diagnosed before pregnancy/Self-reported
Diabetes	Diagnosed before pregnancy/Self-reported
Preterm birth	History of previous preterm birth
Miscarriage	History of previous miscarriage
Heart disease	Diagnosed before pregnancy/Self-reported
*In vitro* fertilization	Fertilization in the current pregnancy
Double/twin pregnancy	Twin pregnancies confirmed by ultrasound
Persistent bleeding	Women presenting persistent bleeding
Severe obesity	Body mass index above 35 kg/m^2^
Heavy smokers	Smoking more than 20 cigarettes a day
Active women	Weekly leisure-time physical activity >150 min/Self-reported

### Logistics and setting

**Fig. 2:**
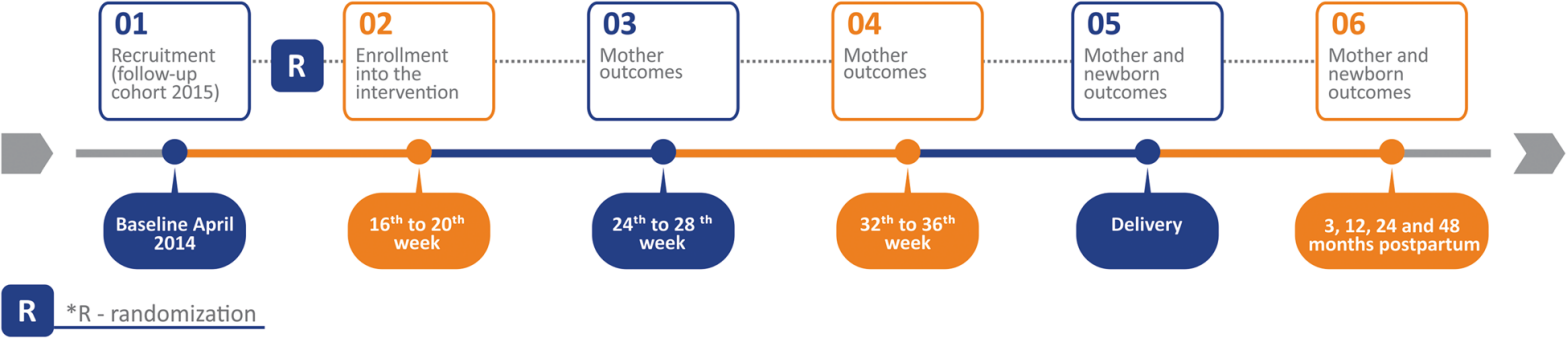
Timeline of PAMELA study

The study begins with one initial contact with the mother, prior to the 16th week of gestation, when interviewers ask for contact information, conduct a brief interview and hand a leaflet explaining the controlled trial. After enrollment in the study, women will be invited to go to the Epidemiology Research Center to collect baseline data, that is, blood and urine sampling, anthropometry, blood pressure and lung function. The same assessments (in both groups) will be repeated at eight and 16 weeks after baseline. The facilities of the Federal University of Pelotas at the Physical Education School will be used for the intervention. A second interview at hospital will take place soon after birth (when children will be measured and mothers will be asked about many aspects of their pregnancy). After these initial data collections, when children are three, 12, 24 and 48 months, interviews will be carried out at the Epidemiologic Research Center or the participant’s household. Fig. 2 shows the recruitment flowchart and subsequent data collections.

### Intervention procedures: exercise protocol

The intervention will start between the 16th and 20th week of gestation and will last 16 weeks. Workouts will be guided and supervised by a team of five previously trained physical education professionals. A set of exercises will be suggested for each workout, involving aerobic activities (treadmill or stationary bike), strength training (dumbbells, machines or elastic bands) and floor exercises. The intensity of the exercises will be set according to each woman’s perceived effort (within the range of 12 to 14 on the Borg Scale) [25], and will be altered according to the progress of pregnancy. The workload will change according to the week of the intervention, and the stages are described in Table 2.

**Table 2:** Stages of training routines during pregnancy according to week of intervention

Weeks of intervention	Warm-up	Aerobic exercise	Strength training/Floor exercises	Stretching
1 to 4, Stage 1	5 min	15 min	35 min (sets: 3 × 12 repetitions)	5 min
5 to 10, Stage 2	5 min	20 min	30 min (sets: 3 × 10 repetitions)	5 min
11 to end, Stage 3	5 min	25 min	25 min (sets: 3 × 8 repetitions)	5 min

Each session will last around 60 min, and will include aerobic exercises (treadmill or stationary cycling), strength training (with focus on major muscle groups and pregnancy-specific exercises to help alleviate low back pain, and work abdominal and pelvic floor muscles to prevent urinary incontinence) and will end with stretching exercises. The exercises are described in the Table 3.

**Table 3:** Description of exercises included in the intervention during pregnancy

Exercise type	Workout description
Aerobic exercise	Treadmill or stationary cycling (unless cycling feels uncomfortable with pregnancy evolution).
	Intensity of continuous exercise: at the highest comfortable intensity (‘talking intensity’): the intensity will be kept at a level that allows a conversation to be carried out, that is, 12 to 14 on a 6 to 20 Borg Scale of Perceived Exertion. Women will be allowed to jog on the treadmill if desired.
Strength training	Weight machines: shoulder press, bench press, knee extension, seated row and hip adduction.
	Free weights and elastic bands exercises for all large muscle groups.
	Intensity of weight training:
	Stage 1: weights will be as heavy as the woman can lift 12 times;
	Stage 2: weights will be as heavy as the woman can lift 10 times;
	Stage 3: weights will be as heavy as the woman can lift 8 times.
Floor and Swiss ball exercises	Mandatory exercises:
	a) alternating arm/leg raise;
	b) ball squats;
	c) spinal rotation;
	d) Kegel contractions;
	e) spinal flexion (cat stretch);
	f) pelvic tilts on the ball.
Stretching	Stretching exercises will especially focus on the cervical area (neck), low back, lower leg (calves), quadriceps, pectoralis and gluteus region. Passive and active stretching will be used, according to the muscle group being stretched.

### Primary outcome measures

Preterm birth (gestational age below 37 weeks of pregnancy), and pre-eclampsia (blood pressures above 140/90 mmHg and proteinuria above 15.0 mg/dL) will be assessed during pregnancy and soon after birth.

### Secondary outcome measures

Secondary outcomes will be assessed during pregnancy and until children reach the age of 48 months, specifically: blood lipid profile, lung function (peak expiratory flow - nSpire Health PiKo-1® - nSpire Health, Inc. - 1830 Lefthand Circle - Longmont, CO 80501, USA), gestational diabetes (self-reported, by the time of delivery), gestational weight gain (calculated based on pre-gestational weight and weight at admission to deliver), mode of delivery (vaginal or cesarean section), birth weight (in grams), length at birth (in cm) and fetal growth (according to the new Intergrowth-21st fetal growth curves [26]). At three months post-partum two outcomes will be measured: post-partum weight retention (difference between current weight and pre-gestational weight) and depression (measured by the Edinburgh Postnatal Depression Scale). Infant neurodevelopment will be assessed (measured by the Battelle Developmental Inventory) at the 12, 24 and 48-month visits [27].

### Follow-up and compliance to the study

To improve the follow-up rate, at the beginning of the study participants will be informed of the importance to attend all sessions, and the staff will register name and contact information for follow-up on missed sessions to collect information on the reasons for the absence, and to offer women the opportunity to attend the session on a different day and/or time as soon as possible. Door-to-door transportation and a kit, containing a t-shirt, running tights and running shoes, will be offered for women in the intervention group. Both groups will be given study t-shirts and laboratory results around 10 days after collection. To be considered adherent to the intervention, women should attend 16 weeks of the program and a minimum of 34 (70 %) workout sessions, and cannot miss more than six training sessions in a row.

### Control group

Women allocated to the control group will be instructed to continue their usual routines, will do the same assessments as the intervention group and will be followed by the 2015 Pelotas (Brazil) Birth Cohort Study.

### Sample size calculation

For the sample size calculation, based on a statistical power of 80 %, a level of significance set at 5 % and using different outcomes from the last birth cohort [28] and estimates based on secular trends (preterm birth at 16 %, gestational hypertension at 18 %, leisure-time physical activity during pregnancy at 13 % and estimate of risk reduction with the intervention at 30 %), we estimated that 213 women would be necessary for the intervention group. The intervention:control ratio will be 1:2, therefore 426 will be included as the control group.

### Statistical analysis

Statistical analyses will be conducted on an intention-to-treat basis, but secondary analyses will be performed, including only those considered adherent to the protocol. Baseline characteristics will be presented using descriptive statistics to compare both groups.

According to the type and distribution of variables, between-groups differences will be evaluated using adequate tests. Continuous variables will be analyzed by t tests (for symmetrically distributed data), or Mann–Whitney U tests (for asymmetrical data). Categorical variables will be analyzed with chi-square tests or Fisher’s exact test, as appropriate. General linear model or logistic regression will be employed to control for confounding factors. More specifically, based on the perinatal study information, preterm birth, gestational diabetes and eclampsia incidences will be compared in the two groups using chi-square tests, followed by multivariable analyses when adequate; linear regressions will be carried out to evaluate potential differences in birth weight, gestational weight gain and lipid profile. Analysis will be carried out in the statistical package STATA 12.0 (StataCorp, 4905 Lakeway Drive. College Station, TX, 77845 USA) and significance will be set at 5 %.

## Discussion

Despite current evidence, few women are physically active during pregnancy in Brazil and worldwide [19, 29, 30]. As physical activity is considered a behavioral aspect of living, changes are not easily made, despite knowledge accumulation. During pregnancy, literature shows that even those women who were previously active decrease their activities, or even quit exercising [31]. This is the first large exercise intervention during pregnancy in Brazil, especially using a population-based sample. If our hypothesis is confirmed, the results of the study will be potentially used routinely to counsel pregnant women during prenatal care about physical activity during pregnancy.

Among the strengths of our study, we highlight that several efforts will be made to improve attendance to the program, such as free transportation to and from the intervention setting and material incentives (athletic apparel from the study organization). Also, our intervention program includes three weekly sessions of exercise, with a large sample size, which is not commonly found in the literature. The main limitation of any behavioral controlled trial is with respect to the exposure, especially in the control group, because we cannot guarantee that these women will not be exposed in their daily lives to some level of physical activity. However, based on previous data from this population, we know that the prevalence of regular leisure-time physical activity during pregnancy is extremely low (below 15 %) and, as we excluded from the randomization women who were previously active, we believe that the number of active women in the control group will not bias the study or lead to any kind of misclassification.

## Trial status

Participant recruitment for this trial is ongoing. Recruitment began on September, 2014 and is expected to end by September, 2015.
